# Supplementary figures and images for: Engaging biological oscillators through second messenger pathways permits emergence of a robust gastric slow-wave during peristalsis
Source: PLoS Comput Biol. 2021 Dec 6;17(12):e1009644. doi: 10.1371/journal.pcbi.1009644 (PMC8675931; doi:10.1371/journal.pcbi.1009644)

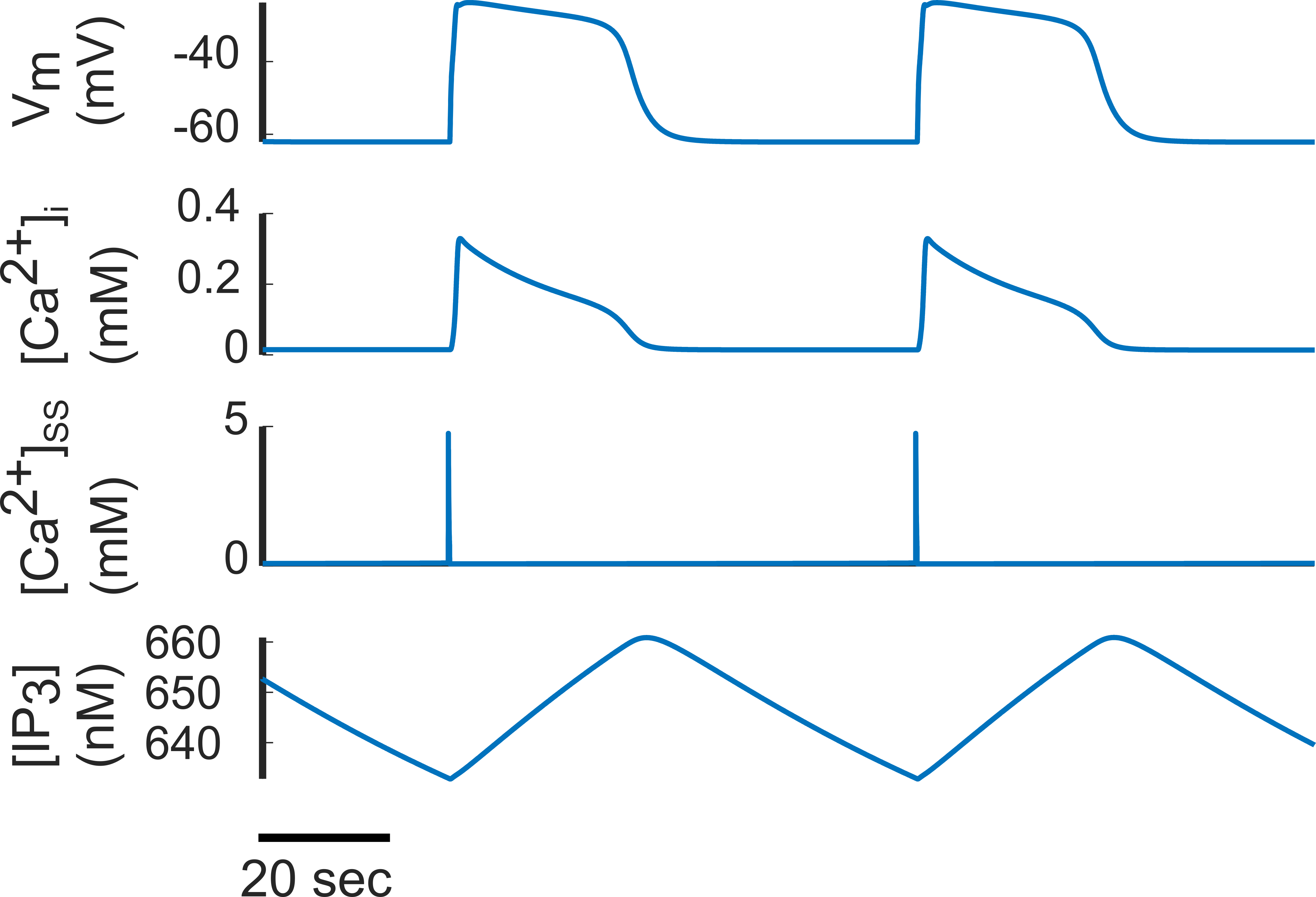

Supplement: S1 Fig — The 4 key state variables for an ICC-SM oscillatory unit have been plotted for 2 representative cycles. The state variables are: membrane potential of an ICC (Vm), intracellular Ca2+ ([Ca2+]i), submembrane space Ca2+ ([Ca2+]SS), and intracellular IP3 concentration ([IP3]). (TIF) [file pcbi.1009644.s001.tif]

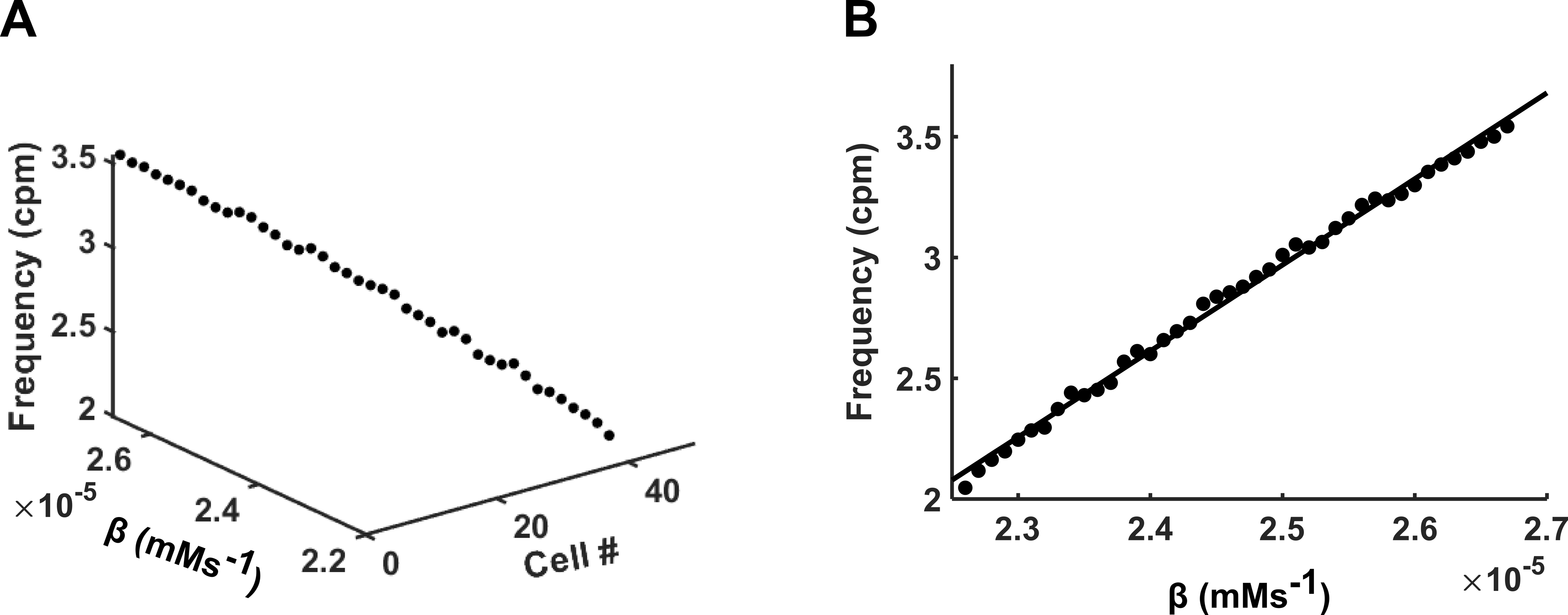

Supplement: S2 Fig — (A) Intrinsic frequency of all 42 OUs have been plotted against the individual OU and its corresponding neural innervation. Intrinsic frequency of 42 OUs have been measured in cycles per minute (cpm) for the corresponding enteric neural stimulus received. (B) Intrinsic frequency of an oscillatory unit is linearly dependent on the enteric neural innervation, β. The best-fit line follows a linear equation of the form: y = Ax+B, where A = 3.563e5, B = −5.937; R2 = 0.9959. (TIF) [file pcbi.1009644.s002.tif]

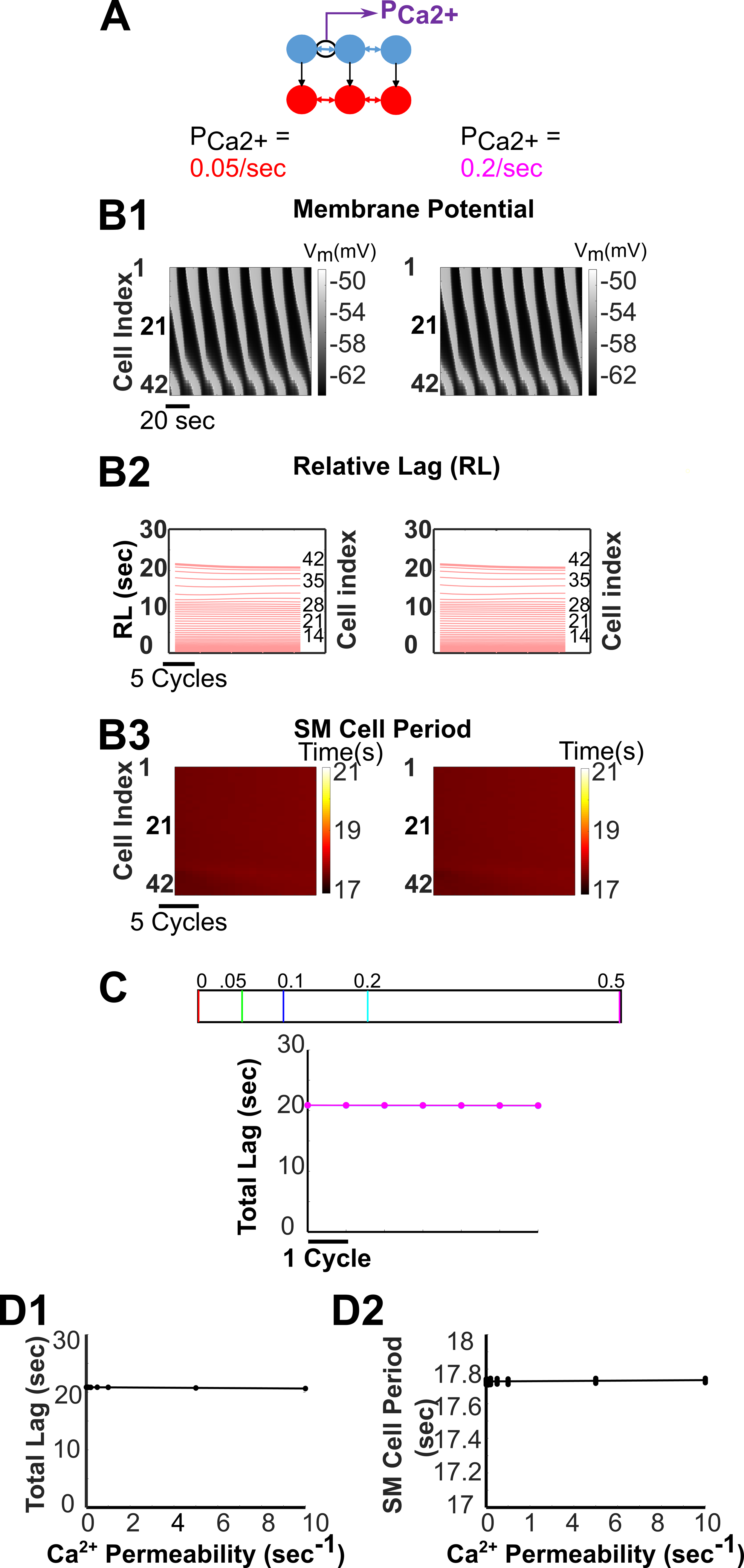

Supplement: S3 Fig — (A) The network. (B) Spatiotemporal map of membrane potential (B1), Relative Lags (B2), and spatiotemporal map of SM Cell Periods (B3) for the network when PCa2+ = 0.05 sec-1 (left panel diagrams) and 0.2 sec-1 (right panel diagrams). (C) The Total Lags for changes in PCa2+ are shown for the last 7 cycles of 900-sec simulations. The corresponding values of these permeabilities in sec-1 are shown in the legend. (D) For several networks, the mean Total Lag (D1) and the SM Cell Period (D2) of the last 7 cycles for each network with respect to its PCa2+ are fit by an approximately constant line. (TIF) [file pcbi.1009644.s003.tif]

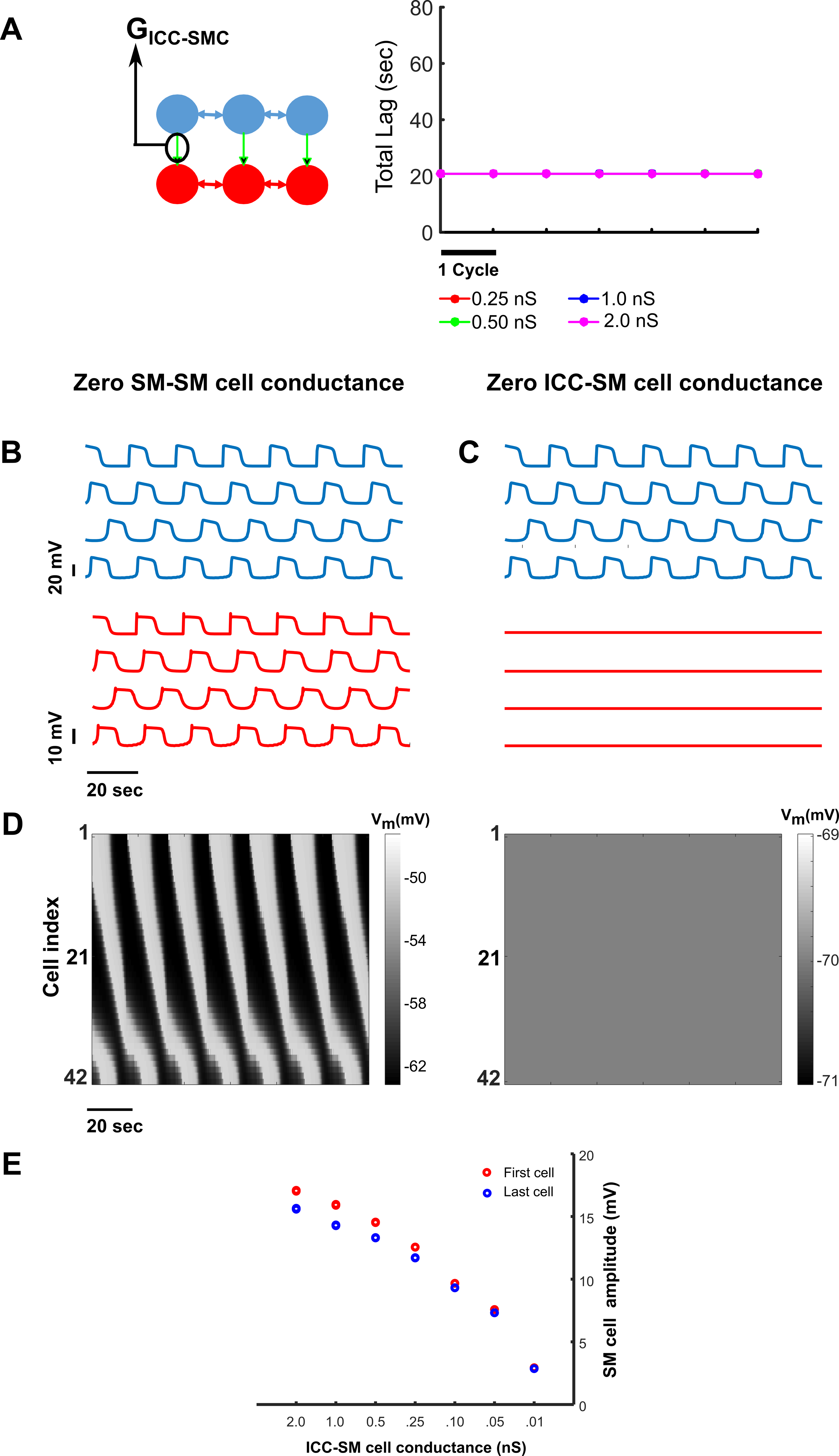

Supplement: S4 Fig — (A) ICC-SM cell electrical conductance does not have any effect on network entrainment evident from the approximately equal values of Total Lag measured for 4 different values of ICC-SM cell electrical conductances. (B) Membrane potential of 4 equidistant ICCs (top diagrams) and SM cells (bottom panel diagrams) in the 42-cell network when ICC-SM cell conductance is 0.5 ns (left) and 0 ns (right). (C) Spatiotemporal map of membrane potential of all 42 SM cells of the network, where ICC-SM cell conductance is 0.5 nS (left) and 0 nS (right). (D) Reduction of ICC to SM cell gap junction conductance reduces the amplitude of SM cell membrane potentials. For representation purposes, here the amplitudes (the peak-to-valley) of membrane potentials of the first and last SM cells of the network are shown. (TIF) [file pcbi.1009644.s004.tif]
