# Supplementary material for: Engaging biological oscillators through second messenger pathways permits emergence of a robust gastric slow-wave during peristalsis
Source: PLoS Comput Biol. 2021 Dec 6;17(12):e1009644. doi: 10.1371/journal.pcbi.1009644 (PMC8675931; doi:10.1371/journal.pcbi.1009644)
Supplement: S1 Table — (DOCX) [file pcbi.1009644.s005.docx]

**S1 Table. Summary of published models for a gastric slow-wave in the stomach.**

| **Model** | **Nature of modeling** | **Single ICC** | **Single SM cell** | **ICC chain(1-D)** | **ICC+SM cell network (2-D)** | **Enteric neuron network** | **Comparison with**  **Experimental data** | | | **IP_3_ Dynamics** | **Gap junction connectivity** |
| --- | --- | --- | --- | --- | --- | --- | --- | --- | --- | --- | --- |
|  |  |  |  |  |  |  | Animal model | Intact freq. | Intrinsic freq. |  |  |
| Corrias & Buist, 2008 | Biophysical | **√** | **×** | **×** | **×** | **×** | Guinea-pig antrum | N/A | **√** | N/A | N/A |
| Corrias & Buist, 2007 | Biophysical | **×** | **√** | **×** | **×** | **×** | Canine antrum SMC | N/A | **√** | N/A | N/A |
| Peng Du, 2010 | Biophysical | **√** | **×** | **√** | **×** | **×** | Simulated in mouse jejunum | N/A | N/A | Dynamic IP_3_ | Electrical |
| Buist, 2010 | Biophysical | **√** | **√** | **√** | **√** | **×** | Guinea-pig stomach SMC | **×** | Antrum- **(√)**  Corpus-**(×)** | Static IP_3_ | Electrical |
| Aliev, 2000 | Coupled chain oscillator | **√** | **√** | **√** | **√** | **×** | Canine intestine | **√** | **√** | N/A | Electrical |
| Edwards, 2006 | Electrical | **√** | **√** | **√** | **√** | **×** | Guinea-pig antrum SMC | N/A | **√** | N/A | Electrical |
| Barth, 2017 | Electrical+ Biophysical | **√** | **√** | **√** | **√** | **√** | Rat colon | N/A | N/A | N/A | Electrical, synaptic and neuromuscular |
| Van Helden, 2003 | Lumped biophysical | **√** | **×** | **√** | **×** | **×** | Guinea-pig pylorus SMC | N/A | N/A | Dynamic IP_3_ | Electrical & Chemical (Ca^2+^ & IP_3_) |
| Our model | Biophysical | **√** | **√** | **√** | **√** | **×** | Guinea-pig & Cat | **√** | Antrum- **(√)**  Corpus-**(√)** | Dynamic IP_3_ | Electrical & Chemical (Ca^2+^ & IP_3_) |
