# Supplementary material for: Engaging biological oscillators through second messenger pathways permits emergence of a robust gastric slow-wave during peristalsis
Source: PLoS Comput Biol. 2021 Dec 6;17(12):e1009644. doi: 10.1371/journal.pcbi.1009644 (PMC8675931; doi:10.1371/journal.pcbi.1009644)
Supplement: S2 Table — (DOCX) [file pcbi.1009644.s006.docx]

**S2 Table. Comparison of our model results to those from specific animal preparations**

|  | **ICC frequency** | **SM cell frequency** | **Stomach frequency** |
| --- | --- | --- | --- |
| **Our model** | ~ 3 cpm | ~ 3 cpm | ~ 3.6 cpm |
| **Guinea-pig** | ~ 3 cpm [5] | ~ 3 cpm [14] |  |
| **Cat** |  |  | ~ 3.6 cpm [39] |
| **Dog** |  |  | ~ 3.7 cpm [40] |
