# Supplementary material for: Engaging biological oscillators through second messenger pathways permits emergence of a robust gastric slow-wave during peristalsis
Source: PLoS Comput Biol. 2021 Dec 6;17(12):e1009644. doi: 10.1371/journal.pcbi.1009644 (PMC8675931; doi:10.1371/journal.pcbi.1009644)
Supplement: S3 Table — Mean ± standard deviation for the last 7 cycles in each simulation (Fig 5D1). The asterisk represents the GICC−ICC value used in the default network in Fig 3. (DOCX) [file pcbi.1009644.s007.docx]

**S5 Table. *Total Lag* and Velocity under different values of** $G_{CICC-IC}$**.** Mean ± standard deviation for the last 7 cycles in each simulation **(Fig 5D1).** The asterisk represents the $G_{ICC-ICC}$value used in the default network in **Fig 3.**

| **G_ICC-ICC_ value (nS)** | ***Total Lag* (sec)** | **Velocity (cm/sec)** |
| --- | --- | --- |
| 0.35 | 53.77 ± 3.63 | N/A |
| 0.53 | 21.99 ± 0.02 | *~0.27* |
| 0.7* | 20.85 ± 0.02 | *~0.29* |
| 1.05 | 19.27 ± 0.03 | *~0.31* |
| 1.4 | 18.22 ± 0.03 | *~0.33* |
| 2.0 | 16.93 ± 0.03 | *~ 0.35* |
| 3.0 | 15.51 ± 0.03 | *~0.39* |
| 5.0 | 13.81 ± 0.03 | *~0.43* |
