# Supplementary material for: Engaging biological oscillators through second messenger pathways permits emergence of a robust gastric slow-wave during peristalsis
Source: PLoS Comput Biol. 2021 Dec 6;17(12):e1009644. doi: 10.1371/journal.pcbi.1009644 (PMC8675931; doi:10.1371/journal.pcbi.1009644)
Supplement: S4 Table — Mean ± standard deviation for the last 7 cycles in each simulation. (DOCX) [file pcbi.1009644.s008.docx]

**S6 Table. *SM Cell Period* under different values of** $G_{ICC-ICC}$**.** Mean ± standard deviation for the last 7 cycles in each simulation.

| **G_ICC-ICC_ value (nS)** | **SM_1_ Period (sec)** | **SM_42_ Period (sec)** |
| --- | --- | --- |
| 0.35 | 17.75 ± 0.01 | 19.41 ± 0.44 |
| 0.53 | 17.75 ± 0.01 | 17.74 ± 0.03 |
| 0.7 | 17.74 ± 0.01 | 17.73 ± 0.01 |
| 1.05 | 17.70 ± 0.01 | 17.69 ± 0.01 |
| 1.4 | 17.67 ± 0.01 | 17.66 ± 0.01 |
| 2.0 | 17.64 ± 0.01 | 17.62 ± 0.01 |
| 3.0 | 17.59 ± 0.01 | 17.58 ± 0.01 |
| 5.0 | 17.52 ± 0.01 | 17.51 ± 0.01 |
