# Supplementary material for: Engaging biological oscillators through second messenger pathways permits emergence of a robust gastric slow-wave during peristalsis
Source: PLoS Comput Biol. 2021 Dec 6;17(12):e1009644. doi: 10.1371/journal.pcbi.1009644 (PMC8675931; doi:10.1371/journal.pcbi.1009644)
Supplement: S5 Table — Mean ± standard deviation for the last 7 cycles in each simulation (Fig 5H1). The asterisk represents the PIP3 value used in the default network in Fig 3. (DOCX) [file pcbi.1009644.s009.docx]

**S7 Table. *Total Lag* and Velocity under different values of** $P_{IP3}$**.** Mean ± standard deviation for the last 7 cycles in each simulation **(Fig 5H1).** The asterisk represents the $P_{IP3}$value used in the default network in **Fig 3.**

| **P_IP3_ value (sec^-1^)** | ***Total Lag* (sec)** | **Velocity (cm/sec)** |
| --- | --- | --- |
| 4.0 | 69.56 ± 2.45 | *N/A* |
| 6.0 | 53.99 ± 4.03 | *N/A* |
| 8.0* | 20.85 ± 0.02 | *~0.29* |
| 12.0 | 16.85 ± 0.03 | *~0.36* |
| 16.0 | 13.17 ± 0.03 | *~0.46* |
| 20.0 | 10.92 ± 0.02 | *~0.55* |
| 30.0 | 8.90 ± 0.01 | *~0.67* |
| 40.0 | 7.93 ± 0.03 | *~0.76* |
