# Supplementary material for: Engaging biological oscillators through second messenger pathways permits emergence of a robust gastric slow-wave during peristalsis
Source: PLoS Comput Biol. 2021 Dec 6;17(12):e1009644. doi: 10.1371/journal.pcbi.1009644 (PMC8675931; doi:10.1371/journal.pcbi.1009644)
Supplement: S6 Table — Mean ± standard deviation for the last 7 cycles in each simulation. (DOCX) [file pcbi.1009644.s010.docx]

**S8 Table. *SM Cell Period* under different values of** $P_{IP3}$**.** Mean ± standard deviation for the last 7 cycles in each simulation.

| **P_IP3_ value (sec^-1^)** | **SM_1_ Period (sec)** | **SM_42_ Period (sec)** |
| --- | --- | --- |
| 4.0 | 17.31 ± 0.01 | 18.44 ± 0.34 |
| 6.0 | 17.53 ± 0.02 | 19.31 ± 0.59 |
| 8.0 | 17.74 ± 0.01 | 17.73 ± 0.01 |
| 12.0 | 17.94 ± 0.01 | 17.92 ± 0.01 |
| 16.0 | 18.31 ± 0.01 | 18.30 ± 0.01 |
| 20.0 | 18.58 ± 0.01 | 18.57 ± 0.01 |
| 30.0 | 18.69 ± 0.02 | 18.69 ± 0.01 |
| 40.0 | 18.71 ± 0.03 | 18.69 ± 0.02 |
