# Supplementary material for: Engaging biological oscillators through second messenger pathways permits emergence of a robust gastric slow-wave during peristalsis
Source: PLoS Comput Biol. 2021 Dec 6;17(12):e1009644. doi: 10.1371/journal.pcbi.1009644 (PMC8675931; doi:10.1371/journal.pcbi.1009644)
Supplement: S1 Text — (PDF) [file pcbi.1009644.s011.pdf]

Engaging Biological Oscillators through Second Messenger Pathways Permits Emergence of a Robust Gastric Slow-Wave during Peristalsis

Short Title: Emergence of Robust Gastric Slow-Wave during Peristalsis

Md Ashfaq Ahmed[1], Sharmila Venugopal[2*], Ranu Jung[1*]

[1]Department of Biomedical Engineering, Florida International University, Miami, Florida, United States of America.

[2]Integrative Biology and Physiology, University of California Los Angeles, Los Angeles, California, United States of America.

* Corresponding authors

E-mail: RJung@fiu.edu; vsharmila@ucla.edu

S1 Text: Model Equations

ICC equations

Environment

$F\_RT = \frac{F}{RT}$

$RT\_F = \frac{RT}{F}$

$T_{correction\_Ca} = Q10Ca^{\frac{Temp - T_{exp}}{10}}$

$T_{correction\_K} = Q10K^{\frac{Temp - T_{exp}}{10}}$

$T_{correction\_Na} = Q10Na^{\frac{Temp - T_{exp}}{10}}$

$T_{correction\_BK} = 1.1(Temp - T_{exp})$

ICC membrane

$V_{cyto} = VolP_{cyto}$

$C_{m,ICC}\frac{dV_{ICC}}{dt} = -(I_{ion,ICC} + I_{g,ICC})$

$I_{ion,ICC} = I_{Na} + I_{L-type} + I_{VDDR} + I_{kv} + I_{ERG} + I_{BK} + I_{CaCl} + I_{NSCC} + I_{kb} + J_{PMCA}FV_{cyto}$

$$\frac{d[Ca^{2+}]_i}{dt} = f_c\left(\frac{-I_{L-type} - I_{VDDR}}{FV_{cyto}} + J_{leak} - J_{PMCA} + J_{g,Ca^{2+}}\right)$$

L-type Ca^{2+} current

$$d_{\infty\_L-type} = \frac{1}{1 + e^{\frac{V_{ICC}+17}{-4.3}}}$$

$tau_{d_{L-type}} = T_{correction_{Ca}}0.001$

$$\frac{d(d_{L-type})}{dt} = \frac{d_{\infty\_L-type} - d_{L-type}}{tau_{d_{L-type}}}$$

$$f_{\infty\_L-type} = \frac{1}{1 + e^{\frac{V_{ICC}+43}{8.9}}}$$

$tau_{f_{L-type}} = T_{correction_{Ca}}0.086$

$$\frac{d(f_{L-type})}{dt} = \frac{f_{\infty\_L-type} - f_{L-type}}{tau_{f_{L-type}}}$$

$$fCa_{\infty\_L-type} = 1 - \frac{1}{1 + e^{-\frac{\Delta[Ca^{2+}]_i - h_{Ca}}{s_{Ca}}}}$$

$tau_{fCa_{L-type}} = T_{correction_{Ca}}0.086$

$$\frac{d(fCa_{L-type})}{dt} = \frac{fCa_{\infty\_L-type} - fCa_{L-type}}{tau_{fCa_{L-type}}}$$

$E_{Ca} = 0.5\ RT\_F\ \ln(\frac{[Ca^{2+}]_o}{[Ca^{2+}]_i})$

$$I_{L-type} = Gmax_{L-type}\, d_{L-type}\, f_{L-type}\, fCa_{L-type}\, (V_{ICC} - E_{Ca})$$
$$J_{PMCA} = \frac{Jmax_{PMCA}}{1 + \frac{0.000298}{[Ca^{2+}]_i}}$$

Voltage-dependent and dihydropyridine-resistant Ca^{2+} current

$$d_{\infty\_VDDR} = \frac{1}{1 + e^{\frac{V_{ICC}+26}{-6.0}}}$$
$$tau_{d_{VDDR}} = T_{correction_{Ca}} 0.006$$
$$\frac{d(d_{VDDR})}{dt} = \frac{d_{\infty\_VDDR} - d_{VDDR}}{tau_{d_{VDDR}}}$$

$$f_{\infty\_VDDR} = \frac{1}{1 + e^{\frac{V_{ICC}+66}{6.0}}}$$
$$tau_{f_{VDDR}} = T_{correction_{Ca}} 0.04$$
$$\frac{d(f_{VDDR})}{dt} = \frac{f_{\infty\_VDDR} - f_{VDDR}}{tau_{f_{VDDR}}}$$

$$I_{VDDR} = Gmax_{VDDR}\, d_{VDDR}\, f_{VDDR}\, (V_{ICC} - E_{Ca})$$

Chloride current

$$d_{\infty\_CaCl} = \frac{1}{1 + (\frac{0.00014}{[Ca^{2+}]_i})^3}$$
$$\frac{d(d_{CaCl})}{dt} = \frac{d_{\infty\_CaCl} - d_{CaCl}}{tau_{d_{CaCl}}}$$
$$E_{Cl} = RT\_F \ln(\frac{[Cl]_i}{[Cl]_o})$$
$$I_{CaCl} = Gmax_{CaCl}\, d_{CaCl}\, (V_{ICC} - E_{Cl})$$

Large conductance Calcium-dependent K^+ current

$$d_{BK} = \frac{1}{1 + e^{\frac{V_{ICC}}{K_{BK}} - (h_{BK} \ln(\frac{[Ca^{2+}]_i}{0.001}))}}$$
$$E_K = RT\_F \ln(\frac{[K]_o}{[K]_i})$$
$$I_{BK} = (Gmax_{BK} + T_{correction\_BK})\, d_{BK}\, (V_{ICC} - E_K)$$

Background K^+ current

$$I_{kb} = Gmax_{kb}\, (V_{ICC} - E_K)$$

Ether-a-go-go (ERG) K^+ channel current

$$d_{\infty\_ERG} = 0.2 + \frac{0.8}{1 + e^{\frac{V_{ICC}+20}{-1.8}}}$$
$$tau_{d_{ERG}} = T_{correction_K} 0.003$$

$$\frac{d(d_{ERG})}{dt} = \frac{d_{\infty\_ERG} - d_{ERG}}{tau_{d_{ERG}}}$$
$$I_{ERG} = Gmax_{ERG}\, d_{ERG}\, (V_{ICC} - E_K)$$

Kv1.1-type K^+ current

$$d_{\infty\_kv} = \frac{1}{1 + e^{\frac{V_{ICC}+25}{-7.7}}}$$
$$tau_{d_{kv}} = T_{correction_K} 0.005$$
$$\frac{d(d_{kv})}{dt} = \frac{d_{\infty\_kv} - d_{kv}}{tau_{d_{kv}}}$$

$$f_{\infty\_kv} = 0.5 + \frac{0.5}{1 + e^{\frac{V_{ICC}+44.8}{4.4}}}$$
$$tau_{f_{kv}} = T_{correction_K} 0.005$$
$$\frac{d(f_{kv})}{dt} = \frac{f_{\infty\_kv} - f_{kv}}{tau_{f_{kv}}}$$
$$I_{kv} = Gmax_{kv}\, d_{kv}\, f_{kv}\, (V_{ICC} - E_K)$$

Na^+ current

$$d_{\infty\_Na} = \frac{1}{1 + e^{\frac{V_{ICC}+47}{-4.8}}}$$
$$tau_{d_{Na}} = T_{correction_{Na}} 0.003$$
$$\frac{d(d_{Na})}{dt} = \frac{d_{\infty\_Na} - d_{Na}}{tau_{d_{Na}}}$$

$$f_{\infty\_Na} = \frac{1}{1 + e^{\frac{V_{ICC}+78}{7.0}}}$$
$$tau_{f_{Na}} = T_{correction_{Na}} 0.0016$$
$$\frac{d(f_{Na})}{dt} = \frac{f_{\infty\_Na} - f_{Na}}{tau_{f_{Na}}}$$

$$E_{Na} = RT\_F \ln(\frac{[Na]_o}{[Na]_i})$$
$$I_{Na} = Gmax_{Na}\, d_{Na}\, f_{Na}\, (V_{ICC} - E_{Na})$$

Non-selective cation current

$$d_{\infty\_NSCC} = \frac{1}{1 + (\frac{K_{NSCC}}{[Ca^{2+}]_{SS}})^{h_{NSCC}}}$$
$$\frac{d(d_{NSCC})}{dt} = \frac{d_{\infty\_NSCC} - d_{NSCC}}{tau_{d_{NSCC}}}$$

$$E_{NSCC} = RT\_F \ln(\frac{[K]_o + [Na]_o\, Na\_K_{perm}}{[K]_i + [Na]_i\, Na\_K_{perm}})$$

$$I_{NSCC} = Gmax_{NSCC}\, d_{NSCC}\, (V_{ICC} - E_{NSCC})$$

Submembrane space

$$V_{mito} = VolP_{mito}$$

$$V_{SS} = VolP_{SS}$$

$$V_{ER} = VolP_{ER}$$

$$J_{ERout} = (Jmax_{IP3} \left(\frac{IP3}{IP3 + d_{IP3}}\right)^3 \left(\frac{[Ca^{2+}]_{SS}}{[Ca^{2+}]_{SS} + d_{ACT}}\right)^3 h^3 + J_{ERleak})([Ca^{2+}]_{ER} - [Ca^{2+}]_{SS})$$

$$J_{SERCA} = \frac{Jmax_{SERCA}{[Ca^{2+}]_{SS}}^2}{{[Ca^{2+}]_{SS}}^2 + {k_{SERCA}}^2}$$

$$MWC = \frac{\frac{conc_{[Ca^{2+}]_{SS}}}{k_{trans}}\,(1 + \frac{[Ca^{2+}]_{SS}}{k_{trans}})^3}{(1 + \frac{[Ca^{2+}]_{SS}}{k_{trans}})^4 + \frac{L}{(1 + \frac{[Ca^{2+}]_{SS}}{k_{act}})^{na}}}$$

$$J_{uni} = \frac{Jmax_{uni}(MWC - ([Ca^{2+}]_m e^{-2FR\_T(\Delta\psi - \Delta\psi_{star})}))\; 2FR\_T(\Delta\psi - \Delta\psi_{star})}{1 - e^{-2FR\_T(\Delta\psi - \Delta\psi_{star})}}$$

$$J_{NaCa} = \frac{Jmax_{NaCa}\, e^{bFR\_T(\Delta\psi - \Delta\psi_{star})}}{(1 + \left(\frac{K\_Na}{[Na]_i}\right)^n)(1 + \frac{K\_Ca}{[Ca^{2+}]_m})}$$

$$J_{leak} = Jmax_{leak}([Ca^{2+}]_{SS} - [Ca^{2+}]_i)$$

$$A_{res} = RT\_F \ln(\frac{K_{res}\sqrt{NADH_m}}{\sqrt{NAD_m}})$$

$$J_o = \frac{rho_{res} 0.5((ra10^{6\,\Delta pH} + rc1e^{6\,\Delta\psi_B FR\_T})e^{A_{res}\,FR\_T} + (-1)rae^{g6FR\_T_{\Delta\psi}} + rc2e^{FR\_T\,A_{res}}\, e^{FR\_T\,\Delta\psi\,6g}))}{(1 + e^{FR\_T\,A_{res}})e^{FR_T\,\Delta\psi_{B6}}(r2 + r3e^{FR\_T\,A_{res}})e^{FR\_T\,\Delta\psi\,6g}}$$

$$J_{Hres} = \frac{rho_{res}\, 3.966\, (ra10^{6\,\Delta pH}\, e^{FR\_T A_{res}} + rb\, 10^{6\,\Delta pH} + (-1)(ra+rb)e^{FR\_T\,\Delta\psi\,6})}{(1 + r1e^{FR\_T A_{res}})e^{6\,\Delta\psi_B FR\_T} + (r2 + r3e^{FR\_T\,A_{res}})\, e^{FR\_T\,\Delta\psi\,6g}}$$

$$J_{glytotal} = \frac{beta_{max}(1 + beta1\, Glc)\, beta2\, Glc\, ATP_i}{1 + beta3ATP_i + (1 + beta4ATP_i)beta5\, Glc + (1 + beta6ATP_i)beta7\, Glc}$$

$$f_{PDHA} = \frac{1}{1 + u2(1 + \frac{u1}{(1 + \frac{[Ca^{2+}]_m}{KCa_{PDH}})^2})}$$

$$J_{red} = J_{red\_basal} + 6.3944\, f_{PDHA}\, J_{glytotal}$$

$$J_{pTCA} = \frac{J_{red\_basal}}{3} + 0.84\, f_{PDHA}\, J_{glytotal}$$

$$A_{F1} = RT\_F \ln(\frac{K_{F1}ATP_m}{ADP_{m\_free}Pi_m})$$

$$J_{pF1} = \frac{rho_{F1}(-1)\left((pa10^{3\,\Delta pH} + pc1e^{3\,\Delta\psi_B FR\_T})e^{A_{F1}\,FR\_T} + (-1)pae^{3\,\Delta\psi FR\_T}\right) + pc2e^{FR\_T\,A_{F1}}\, e^{3FR\_T\,\Delta\psi}}{(1 + p1e^{FR\_T A_{F1}})e^{3\,\Delta\psi_B FR\_T} + (p2 + p3e^{FR\_T\,A_{F1}})\, e^{3FR\_T\,\Delta\psi}}$$

$$J_{HF1} = \frac{(-1)rho_{F13}\, (pa10^{3\,\Delta pH}\, e^{FR\_T A_{F1}} + pb\, 10^{3\,\Delta pH} + (-1)(pa+pb)e^{3\,FR\_T\,\Delta\psi})}{(1 + p1e^{FR\_T A_{F1}})e^{3\,\Delta\psi_B FR\_T} + (p2 + p3e^{FR\_T\,A_{F1}})\, e^{3FR\_T\,\Delta\psi}}$$

$$J_{ANT} = \frac{Jmax_{ANT}(1-(\frac{ATP4_i ADP3m}{ADP3_i ATP4m} e^{(-1)FR\_T\,\Delta\psi}))}{(1+\frac{ATP4_i}{ADP3_i} e^{(-1)frac\ FR\_T\,\Delta\psi})(1+\frac{ADP3m}{ATP4m})}$$
$$PMF = \Delta\psi - (2.303\ RT\_F\ \Delta pH)$$
$$J_{Hleak} = g_H\ PMF$$
$$J_{pGly} = 0.15\ J_{glyTotal}$$
$$J_{hydSS} = \frac{J_{hydmax}}{1 + (\frac{K_{Glc}}{Glc})^{nhyd}}$$
$$J_{hyd} = K_{hyd} ATP_i + J_{hydSS}$$

$$\frac{d(NADH_m)}{dt} = J_{red} - J_o$$
$$NAD_m = total_{NAD_m} - NADH_m$$
$$\frac{d(ADP_m)}{dt} = J_{ANT} - J_{pTCA} - J_{pF1}$$
$$ATP_m = total_{ANP_m} - ADP_m$$
$$ADP_{mfree} = 0.8 ADP_m$$
$$ADP3_m = 0.45 ADP_{mfree}$$
$$ATP4_m = 0.05 ATP_m$$
$$\frac{d(ADP_i)}{dt} = \frac{-J_{ANT} V_{SS}}{V_{cyto}} + J_{hyd} - J_{pGly}$$
$$ADP_i = total_{ANP_i} - ADP_i$$
$$ADP_{ifree} = 0.3 ADP_i$$
$$ADP3_i = 0.45 ADP_{ifree}$$
$$MgADP_i = 0.55 ADP_{ifree}$$
$$ATP4_i = 0.05 ATP_i$$
$$\frac{d([Ca^{2+}]_{SS})}{dt} = fc(\frac{(J_{NaCa} - J_{Uni})V_{mito}}{V_{SS}} + \frac{(J_{ERout} - J_{SERCA})V_{ER}}{V_{SS}} + \frac{(-J_{leak})V_{cyto}}{V_{SS}})$$

$$\frac{d([Ca^{2+}]_m)}{dt} = fm(J_{uni} - J_{NaCa})$$
$$\frac{d([Ca^{2+}]_{ER})}{dt} = fe(J_{SERCA} - J_{ERout})$$

$$\frac{d(\Delta\psi)}{dt} = \frac{FV_{mito}}{C_{mito}}(J_{Hleak} - J_{Hres} + J_{ANT} + J_{HF1} + 2J_{uni})$$
$$\frac{d(h)}{dt} = \frac{d_{INH} - (h([Ca^{2+}]_{SS} + d_{INH}))}{tau_h}$$
$$\frac{d[IP_3]}{dt} = P_{MV}\left(1 - \frac{V_m^8}{k_v^8 + V_m^8}\right) - \eta[IP3] - V_{m4}\ \frac{[IP_3]^4}{k_4^4 + [IP_3]^4} + \beta + J_{g,IP3}$$

SM cell equations

L-type Ca^{2+} current

$$d_{\infty\_L-typeSM} = \frac{1}{1+e^{\frac{V_{SM}+17}{-4.3}}}$$

$$tau_{d_{L-typeSM}} = 0.0047Q10Ca^{\frac{Temp-T_{exp}}{10}}$$

$$\frac{d(d_{L-typeSM})}{dt} = \frac{d_{\infty\_L-typeSM} - d_{L-typeSM}}{tau_{d_{L-typeSM}}}$$

$$f_{\infty\_L-typeSM} = \frac{1}{1+e^{\frac{V_{SM}+43}{8.9}}}$$

$$tau_{f_{L-typeSM}} = 0.086Q10Ca^{\frac{Temp-T_{exp}}{10}}$$

$$\frac{d(f_{L-typeSM})}{dt} = \frac{f_{\infty\_L-typeSM} - f_{L-typeSM}}{tau_{f_{L-typeSM}}}$$

$$fCa_{\infty\_L-typeSM} = \frac{1}{1+e^{\frac{\Delta[Ca^{2+}]_{iSM}-h_{CaSM}}{s_{CaSM}}}}$$

$$tau_{fCa_{L-type}} = 0.002Q10Ca^{\frac{Temp-T_{exp}}{10}}$$

$$\frac{d(fCa_{L-typeSM})}{dt} = \frac{fCa_{\infty\_L-typeSM} - fCa_{L-typeSM}}{tau_{fCa_{L-typeSM}}}$$

$$E_{Ca\_SM} = 0.5\ RT\_F\ \ln(\frac{[Ca^{2+}]_o}{[Ca^{2+}]_{iSM}})$$

$$I_{L-typeSM} = Gmax_{L-typeSM}\ d_{L-typeSM}\ f_{L-typeSM}\ fCa_{L-typeSM}\ (V_{SM} - E_{Ca\_SM})$$

Low voltage activated Ca^{2+} current

$$d_{\infty\_LVA} = \frac{1}{1+e^{\frac{V_{SM}+27.5}{-10.9}}}$$

$$tau_{d_{LVA}} = 0.003Q10Ca^{\frac{Temp-T_{exp}}{10}}$$

$$\frac{d(d_{LVA})}{dt} = \frac{d_{\infty\_LVA} - d_{LVA}}{tau_{d_{LVA}}}$$

$$f_{\infty\_LVA} = \frac{1}{1+e^{\frac{V_{SM}+15.8}{7.0}}}$$

$$tau_{f_{L-typeSM}} = 0.00758Q10Ca^{\frac{Temp-T_{exp}}{10}}e^{0.00817V_{SM}}$$

$$\frac{d(f_{LVA})}{dt} = \frac{f_{\infty\_LVA} - f_{LVA}}{tau_{f_{LVA}}}$$

$$I_{LVA} = Gmax_{LVA}\ d_{LVA}\ f_{LVA}\ (V_{SM} - E_{Ca\_SM})$$

Delayed rectifier K^{+} current

$$xr1_{\infty} = \frac{1}{1+e^{\frac{V_{SM}+27}{-5.0}}}$$

$$tau_{xr1} = 0.08Q10K_{SM}^{\frac{Temp-T_{exp}}{10}}$$

$$\frac{d(xr1)}{dt} = \frac{xr1_{\infty} - xr1}{tau_{xr1}}$$

$$xr2_{\infty} = \frac{0.8}{1 + e^{\frac{V_{SM}+58}{10.0}}}$$

$$tau_{xr2} = Q10K_{SM}^{\frac{Temp-T_{exp}}{10}}(-0.707 + 1.481e^{\frac{V_{SM}+36}{92}})$$

$$\frac{d(xr2)}{dt} = \frac{xr2_{\infty} - xr2}{tau_{xr2}}$$

$$I_{kr} = Gmax_{kr}\, xr1\, xr2\ (V_{SM} - E_{K\_SM})$$

A-type K^+ current

$$xa1_{\infty} = \frac{1}{1 + e^{\frac{V_{SM}+26.5}{-7.9}}}$$

$$tau_{xa1} = Q10K_{SM}^{\frac{Temp-T_{exp}}{10}}(0.0318 + 0.1751e^{(-0.5)(\frac{V_{SM}+44.4}{22.3})^2})$$

$$\frac{d(xa1)}{dt} = \frac{xa1_{\infty} - xa1}{tau_{xa1}}$$

$$xa2_{\infty} = 0.1 + \frac{0.9}{1 + e^{\frac{V_{SM}+65}{6.2}}}$$

$$tau_{xa2} = 0.09Q10K_{SM}^{\frac{Temp-T_{exp}}{10}}$$

$$\frac{d(xa2)}{dt} = \frac{xa2_{\infty} - xa2}{tau_{xa2}}$$

$$I_{ka} = Gmax_{ka}\, xa1\, xa2\ (V_{SM} - E_{K\_SM})$$

Na^+ current

$$m_{\infty\_Na} = \frac{1}{1 + e^{\frac{V_{SM}+47}{-4.8}}}$$

$$tau_{m_{Na}} = Q10Na_{SM}^{\frac{Temp-T_{exp}}{10}}(-0.000017V_{SM} + 0.00044)$$

$$\frac{d(m_{Na})}{dt} = \frac{m_{\infty\_Na} - m_{Na}}{tau_{m_{Na}}}$$

$$h_{\infty\_Na} = \frac{1}{1 + e^{\frac{V_{SM}+78}{3.0}}}$$

$$tau_{h_{Na}} = Q10Na_{SM}^{\frac{Temp-T_{exp}}{10}}(-0.00025V_{SM} + 0.0055)$$

$$\frac{d(h_{Na})}{dt} = \frac{h_{\infty\_Na} - h_{Na}}{tau_{h_{Na}}}$$

$$I_{Na\_SM} = Gmax_{Na}\, m_{Na}\, h_{Na}\ (V_{SM} - E_{Na\_SM})$$

Non-selective cation current

$$m_{\infty\_NSCC} = \frac{1}{1+e^{\frac{V_{SM}+25}{-20.0}}}$$
$$tau_{m_{NSCC}} = \frac{0.15}{1+e^{\frac{V_{SM}+66}{-26.0}}}$$
$$\frac{d(m_{NSCC})}{dt} = \frac{m_{\infty\_NSCC} - m_{NSCC}}{tau_{m_{NSCC}}}$$
$$h_{Ca} = \frac{1}{1+(\frac{[Ca^{2+}]_{iSM}}{K_{CaNSCC}})^{n_{Ca}}}$$
$$r_{lig} = \frac{1}{1+(\frac{0.01}{ACh})}$$
$$I_{NSCC\_SM} = Gmax_{NSCC\_SM}\, m_{NSCC}\, h_{Ca} r_{lig}\ (V_{SM} - E_{NSCC\_SM})$$

Large conductance Calcium-dependent K+ current

$$d_{BK\_SM} = \frac{1}{1+e^{\frac{V_{SM}}{K_{BK\_SM}} - (h_{BK\_SM}\ln(\frac{[Ca^{2+}]_{iSM}}{Ca_{set}}))}}$$
$$I_{BK\_SM} = (Gmax_{BK\_SM} + 1.1(Temp - T_{exp}))\, d_{BK\_SM}\,(V_{SM} - E_{K\_SM})$$

$$I_{bk\_SM} = Gmax_{bkS\_M}\,(V_{SM} - E_{K\_SM})$$

$$J_{CASR} = 1000 Jmax_{CASR}[Ca^{2+}]_{iSM}{}^{1.34}$$

$$\frac{d[Ca^{2+}]_{iSM}}{dt} = \left(\frac{-I_{L-typeSM} - I_{LVA}}{0.002FV_{SM}} - J_{CASR}\right)$$

SM cell membrane

$$I_{total,SM} = I_{Na\_SM} + I_{L-typeSM} + I_{LVA} + I_{kr} + I_{ka} + I_{BK\_SM} + I_{NSCC\_SM} + I_{bk\_SM}$$

$$I_{coup} = g_{coup}(V_{ICC} - V_{SM})$$

$$\frac{d(V_{SM})}{dt} = \frac{-1}{Cm_{SM}} - \left(I_{total,SM} + I_{g,SM} - I_{coup}\right)$$

Gap junction currents

$$I_{g,ICC} = G\ \textstyle\sum_j (V_i - V_j)$$
$$I_{g,SM} = G_{SM}\ \textstyle\sum_j (V_{iSM} - V_{jSM})$$

$$J_{g,Ca^{2+}} = -P_{Ca2+} \sum_j ([Ca^{2+}]_i - [Ca^{2+}]_j)$$
$$J_{g,IP3} = -P_{IP3} \sum_j \left([IP_3]_i - [IP_3]_j\right)$$
